# Functional Multipotency of Stem Cells: A Conceptual Review of Neurotrophic Factor-Based Evidence and Its Role in Translational Research

**DOI:** 10.2174/157015911798376299

**Published:** 2011-12

**Authors:** Yang D Teng, Dou Yu, Alexander E Ropper, Jianxue Li, Serdar Kabatas, Dustin R Wakeman, Junmei Wang, Maryrose P Sullivan, D. Eugene Redmond, Robert Langer, Evan Y Snyder, Richard L Sidman

**Affiliations:** 1 Division of SCI Research, Veterans Affairs Boston Healthcare System, Boston, MA, USA; 2 Department of Neurosurgery, Harvard Medical School, Brigham & Women’s Hospital, and Children’s Hospital Boston, Boston, MA, USA; 3 Department of Physical Medicine & Rehabilitation, Harvard Medical School, Spaulding Rehabilitation Hospital, Boston, MA, USA; 4 Department of Neurology, Beth Israel-Deaconess Medical Center, Harvard Medical School, Boston, MA, USA; 5 Stem Cell and Regeneration Program, the Sanford-Burnham Medical Research Institute, La Jolla, CA, USA; 6 Department of Urology, VA Boston Health Care System, and Department of Surgery, Harvard Medical School/Brigham & Women’s Hospital, Boston, MA, USA; 7 Departments of Psychiatry and Neurosurgery, Yale University School of Medicine, New Haven, CT, USA; 8 Department of Chemical Engineering, Massachusetts Institute of Technology, Cambridge, MA, USA

**Keywords:** Stem cells, Neural stem cells, Multipotency, Neurotrophic factors, Neural Repair, Spinal Cord Injury.

## Abstract

We here propose an updated concept of stem cells (SCs), with an emphasis on neural stem cells (NSCs). The conventional view, which has touched principally on the essential property of lineage multipotency (e.g., the ability of NSCs to differentiate into all neural cells), should be broadened to include the emerging recognition of biofunctional multipotency of SCs to mediate systemic homeostasis, evidenced in NSCs in particular by the secretion of neurotrophic factors. Under this new conceptual context and taking the NSC as a leading example, one may begin to appreciate and seek the “logic” behind the wide range of molecular tactics the NSC appears to serve at successive developmental stages as it integrates into and prepares, modifies, and guides the surrounding CNS micro- and macro-environment towards the formation and self-maintenance of a functioning adult nervous system. We suggest that embracing this view of the “multipotency” of the SCs is pivotal for correctly, efficiently, and optimally exploiting stem cell biology for therapeutic applications, including reconstitution of a dysfunctional CNS.

## BACKGROUND

Along with refinement of our understanding on the biology and translational potential of neural stem cells (NSCs) [1-6], there has been an increasingly appreciated paradigm shift regarding how the adult mammalian central nervous system (CNS) could be repaired for functional restoration. The long-standing dogma that the adult CNS was immutable to anatomical and functional repair was challenged by an unambiguous demonstration that cells derived from the CNS from a variety of structures and at different developmental stages, including adulthood, possess stem-like properties. NSCs are primordial and uncommitted cells that have been believed to give rise to the vast array of more specialized cells of the CNS. They are operationally defined by their abilities (1) to differentiate into cells of all neural lineages (i.e. neurons of multiple subtypes, oligodendroglia, and astroglia) in multiple regional and developmental contexts (i.e., to be multipotent); (2) to self-renew (i.e., to give rise also to new NSCs with similar potential); (3) to migrate and populate developing and/or degenerating CNS regions; and (4) to have biofunctional multipotency to mediate systemic homeostasis through capacities such as production of trophic factors, formation of gap junctions, etc. [7,8]. Since monoclonal derivation of progeny is obligatory to the definition of NSC: that is, a single cell must possess all the aforementioned attributes, which suggests the ready availability of NSCs, many studies, including ours, in the past two decades, provided hope that the use of NSCs might circumvent some limitations of presently available graft material and gene transfer vehicles and make feasible a broader range of therapeutic strategies. This significant advance has led neurobiologists to speculate how such phenomena might be further harnessed for both therapeutic advantage and for better understanding of developmental neurobiology mechanisms.

Most studies, to date, are focused principally on exploring the biologically most apparent features of NSCs in comparison to existing gene therapy and cell transplantation strategies. For this purpose, NSCs, with their homogeneous and well-defined neural differentiation capacity, have been pursued primarily as a modality that could replace dead or degenerating neural cells in a wide variety of neurological diseases and trauma. For certain pathological models of the brain, NSCs and their progeny not only could integrate with host tissue at their site of implantation [9-11], but also could interact with distant brain tissue *via *migration in response to chemical cues in the host [12-15]. The responsiveness of NSCs to microenvironmental cues in the surrounding CNS tissue provides them with a unique trait distinguishing them from fetal brain/spinal cord tissue and non-neural cells (e.g., fibroblasts or mesenchymal stromal stem cells [MSCs]), as well as from most viral vectors and protein infusion devices for gene delivery. For example, haematopoietic cells could not efficiently cross the blood–brain barrier and integrate throughout the CNS as effectively as NSCs. In addition, NSCs are able to “sense” the presence of degenerating neural tissue [13,14,16-18]. Conversely, effective replacement of dying or dysfunctional neurons by NSCs in the adult CNS, an ideal hypothesis the field initially entertained, has been proven to be a much more challenging endeavor, though in a few experimental models of neurodegeneration such NSC-based cell substitution appeared to be feasible in replacing dead oligodendrocytes [9,19]. We therefore have discussed here the biological perspectives of epigenetic and genetic features of rodent (in particular, murine) and human neural stem cells regarding their capacity to produce neurotrophic factors (NTFs). Also included are views of investigators in the field on how to modulate and/or augment the homeostatic function of NSCs. Finally we summarize our current thoughts about the critical roles that NSCs may play in designs for improved regimens of NTF delivery regarding neural repair and pathology correction.

## NEUROTROPHIC FACTORS AND NEURAL STEM CELLS

Because NSCs are dynamic developmental entities their therapeutic potential has been studied primarily *via *strategies devised to realize desirable neural phenotypic differentiations. Much less recognized and appreciated is the fact that these developmental cells, by innate biology, produce neurotrophic factors that influence the growth and well-being of their own and surrounding tissues *via *autocrine and paracrine mechanisms. Efforts to understand this more recently recognized perspective of stem cell biology were first triggered by the observation that undifferentiated donor NSCs could exert marked neuroprotection to the spinal cord following traumatic injury, engendering a speculation that NSCs and other stem cells may have inherent capability to synthesize trophic factors [20]. Considerable research endeavors have since taken place to seek beneficial impact of stem cells (e.g., NSCs, MSCs, and embryonic stem cells [ESCs]) that are mediated mainly through soluble trophic factors and other cytokines that enable the body to reestablish homeostasis after pathologic and traumatic insults, inflammation, and tissue infarction or degeneration [21-24]. In this article, based on data mainly derived from studies on traumatic spinal cord injury (SCI), we seek to establish conceptually the biological principles of trophic factor delivery by stem cells as a novel approach to new therapeutic strategies based on developmental mechanisms [7]. For this goal, we have expanded the conceptual scope of tropic factors to include the following categories of molecules.

By our definition, neurotrophic factors, here including classic neurotrophins, are groups of naturally occurring substances that support neural cell survival, proliferation, migration, differentiation, growth, and function [25]. They are usually proteins or simpler peptides, and are essential regulating and signaling molecules for cell development and function. Neurotrophic factors contribute to neurogenesis, and are critical in shaping neural network structure and physiological processes such as learning, memory formation, and sensorimotor function [26]. Some neurotrophic factors, conversely, can be directly involved in pathological processes [25], *via *effects on synaptic biology, dynamics of neurotransmitter release and synaptic efficacy, whereas others act through secondary messengers and various kinases [27]. 

Listed below are the six biological families of neurotrophic factors that directly impact stem cell development, and are expressed by natural or bioengineered stem cells. 1. Classic neurotrophins that comprise nerve growth factor (NGF), brain-derived neurotrophic factor (BDNF), neurotrophin-3 (NT-3), and neurotrophin-4 (NT-4); 2. The transforming growth factor (TGF)-beta family, represented by glial cell line-derived neurotrophic factor (GDNF) and the bone morphogenic proteins (BMPs); other members are neurturin, artemin, and persephin; 3. The cytokine growth factor family, including ciliary neurotrophic factor (CNTF), leukemia inhibitory factor (LIF), and cardiotropin-1; 4. The epidermal growth factor (EGF) family, consisting of EGF, transforming growth factor-alpha (TGF-alpha), the neuregulins, and neural and thymus-derived activator for ErbB kinases (NTAK); 5. The insulin-like growth factors (IGFs), consisting of insulin-like growth factor I (IGF-I) and IGF-II; and 6. The fibroblast growth factor (FGF) family, consisting of at least 24 different proteins including acidic FGF (FGF 1) and basic FGF (FGF 2) [28,29]. 

For the purpose of this review, we focus our discussion only on the family of classic neurotrophins since all its members (i.e., NGF, BDNF, NT-3, and NT4/5) have been demonstrated to play critical roles in neuronal survival, differentiation, and/or function in neurotrauma settings [30]. The neurotrophins act by binding to receptor tyrosine kinases of the Trk families. NGF binds for tropomyosin-receptor-kinase-A (TrkA), BDNF and NT-4/5 bind TrkB, and NT-3 preferentially binds TrkC. Importantly, NT-3 up-regulates TrkC expression in NSCs and induces them to differentiate into neurons [31-33]. For example, Ad-TrkC can be constructed to express TrkC effectively in NSCs, enhancing NT-3-mediated neuronal differentiation, an approach that may offer additional strategies for treating neurotrauma [33]. Overexpression of TrkC in rat neural progenitor cells improves survival and reduces glial differentiation of donor cells in the intact spinal cord [34]. Neurotrophin-mediated Trk signaling plays an essential, cell-autonomous role in regulating the proliferation and differentiation of embryonic cortical precursors and thus controls cortical development at early stages [35]. Multiple alternatively spliced isoforms have been observed for TrkA, TrkB, and TrkC, especially in non-neuronal cells [31,36-38]. Some of these isoforms lack the cytoplasmic tyrosine kinase domain, but retain selective signaling and may inhibit neurite outgrowth [37,39-41]. However, brain-derived neurotrophic factor (BDNF) has proliferative effects on NSCs through the truncated TRK-B receptor [42]. Neurotrophins also bind to p75NTR. Activation of this receptor may cause cell death rather than survival, as p75NTR^-/-^ mice show reductions in neuronal cell death after pilocarpine-induced seizures compared to wild-type [43,44]. Interestingly, the p75 neurotrophin receptor is also involved in proliferation of undifferentiated mouse ESCs, and becomes down-regulated upon ES cell differentiation [45]. NT-3 improves the neural differentiation of skin-derived precursors (SKPs) induced by retinoic acid (RA) through a p75NTR-dependent signaling pathway [46,47]. In the following sections, we will discuss how a novel concept of using prototype or engineered NSCs to deliver therapeutic neurotrophic factors was developed, based on data collected from SCI investigations, and how this concept has subsequently been used to guide administration of neurotrophic factors to treat or investigate representative neurological disorders such as SCI, Alzheimer's disease (AD) and amyotrophic lateral sclerosis (ALS).

## ADMINISTRATION OF NEUROTROPHIC FACTORS FOR TREATING SCI

The pharmacological use of neurotrophic factors for SCI treatment has been tested primarily with two neurotrophins: BDNF and NT-3. Unlike peripheral nerve axons, which regenerate well, the adult mammalian spinal cord appears to have an unsuccessful capacity for axonal regrowth [48,49]. Neurotrophic factors, when administered in appropriate physical and temporal gradients, have been shown to support axonal growth after peripheral nerve injury. In contrast, antagonizing these growth factors in the extracellular matrix impedes neurite outgrowth [50,51]. Due to these findings, plus the fact that molecules have been identified that can suppress axonal sprouting and regeneration in the CNS (e.g., the chondroitin sulfate proteoglycan [CSPG] molecules) [52,53], substantial experimental efforts have been given to enhance regeneration of the supraspinal descending axons through activating neurotrophic factor-mediated mechanisms that may overcome the growth inhibitory environment in the CNS [54-60]. Additional approaches include combinatorial tactics that comprise transplantation of progenitor cells into the lesion cavity with simultaneous administration of neurotrophic factors [61], or direct augmentation of key molecules of trophic factor-triggered signal transduction, such as raising intracellular cAMP levels pharmacologically [62,63]. Many of these studies report incremental improvement in axonal growth and/or motor behavior, projecting hope for advancing towards the ultimate goal of restoring function following clinical SCI. 

As examples, several groups reported that BDNF can enhance growth of supraspinal motor axons into permissive growth milieus placed at sites of SCI. Both rubrospinal axons [64,65] and reticulospinal axons [66], descending networks that regulate spinal cord motor neural activities, regenerate in response to BDNF stimulation. Reversal of atrophy of neurons in the red nucleus, even one year after SCI, can occur after BDNF stimulation [67]. However, corticospinal tract (CST) axons did not respond to BDNF post-SCI, even though BDNF prevents the death of corticospinal neuronal cell bodies when directly applied to the cerebral cortex. This outcome has been attributed to the lack of BDNF receptor TrkB on CST fibers [68]. 

The ability of NT-3 treatment to promote regeneration of severed CST axons was also reported [69]. This triggered additional bioengineering initiatives to enhance the delivery efficiency of NT3. Subsequently, Grill and colleagues, adapting a gene therapy strategy, demonstrated that autologous fibroblasts genetically modified to secrete NT-3 could be transplanted into the epicenter of experimental SCI to improve neural repair [70]. More recently, Tuszynski’s group at UCSD reported beneficial effects of artificially established NT-3 gradients using cell-based gene delivery, increasing axonal regeneration in experimental SCI [71]. Importantly, studies comparing the therapeutic effect on axonal growth of three different methods of replenishing neurotrophic factors (direct infusion, transplantation of unmodified cells, and transplantation of genetically engineered cells with enhanced secretory function) have reported favorable results with genetically modified cells [72]. These data support the notion that using cells to administer neurotrophic factors may offer clinically favorable pharmacodynamic properties due to the interactive capability of “these functional basic units of life” [71,72]. Therefore, donor cells such as NSCs, in particular, have become highly promising candidates for delivering NTFs due to their innate biology, which regulates molecule secretion in response to environmental changes in the nervous system [7].

This viewpoint is further strengthened by observation of a therapeutic impact of endogenous NSCs on experimental SCI under the enhanced physical activity paradigm of rehabilitation that induced functional recovery. In some cases, there was a positive correlation between degrees of functional improvement and the number of nestin-expressing NSCs present in the post-lesion spinal cord [73-76], suggesting that NSC proliferation enhanced by exercise may help augment NTF production (see below for more details). Additional support comes from the facts that severe side effects of neurotrophic factors could be caused by conventional drug delivery regimens that lack biological feedback regulations, and by barriers imposed by the peptidergic structure of these trophic factors, which impair their penetration into the brain or spinal cord parenchyma, and therefore make their pharmaco-therapeutic properties difficult to evaluate [77]. 

## A NOVEL CONCEPT OF USING PROTOTYPE OR ENGINEERED NSCS TO DELIVER NEUROTROPHIC FACTORS: INSIGHTS FROM STUDIES OF ENDOGENOUS NSCS AND ACTIVITY-DEPENDENT RECOVERY FOLLOWING SCI

Endogenous NSCs in the spinal cord and their response to trauma: Mitotic activity has been known to persist in the adult mammalian spinal cord [78]. Weiss and colleagues found that the lumbosacral spinal cord normally produces the greatest number of multipotent cells, and the cervical cord the least [79]. Under most conditions, adult NSCs have astroglial characteristics and express glia fibrillary-associated protein (GFAP) [78], but retain the ability to undergo neurogenesis [80,81], and can give rise to functional neurons and glia *in vitro* and *in vivo* [82]. It has been shown that neurotrauma significantly increases the proliferative capacity of spinal cord NSCs. Ependymal zone cells that express low levels of nestin were found to be upregulated, increasing mitotic activity and generating glia, shortly after an injury to the dorsal funiculus of the spinal cord. If implanted in a pro-neurogenesis environment [74,75,83], they are also capable of migrating and undergoing neurogenesis [81,83,84,85]. 

Ependymal and parenchymal neural progenitors were also reported to be activated in response to spinal cord transaction [86,87]. When isolated *in vitro, *they generate astrocytes and oligodendrocytes but few neurons. A fraction of ependymal zone cells possessing stem cell properties or oligodendrocyte progenitor cells in the parenchyma proliferate following compression and contusion injuries [84,88], and appeared to participate in repopulation of the injured areas, suggesting their potential participation in lesion repair [89,90]. This response of NSCs to the injury occurs rather rapidly; for instance, nestin was seen in ependymal cells as early as 1 day after minimal sharp instrument injury to the lateral columns [91]. This injury, however, did not supply a sufficient stimulus for ependymal zone cells to migrate to the lesion site. A relatively larger injury to the dorsal funiculus, by contrast, led to ependymal zone NSC migration from the central canal to the lesion site [82].

Since brain NSCs have been reported to respond to external stimuli such as enhanced physical activity and exercises, by increasing their proliferation, differentiation and survival [92], an extension of that rationale is to ascertain whether physical activity and exercise could serve as an independent stimulus for endogenous NSC proliferation also in the adult spinal cord. *We earlier hypothesized that a major function of proliferating NSCs in the adult mammalian CNS is the production of trophic factors and other homeostasis-maintaining molecules that are essential for endogenous healing and plasticity processes in the spinal cord *[93]. Our published data suggest that physical activity may be beneficial partially by increasing proliferation of endogenous NSCs that produce neurotrophic factors such as GDNF and BDNF, which in turn mediate neuroplasticity and improve sensorimotor functions [74]. Conversely, the contribution of neurotrophic factors to exercise-induced functional recovery *per se* has been investigated extensively. Voluntary exercise induces an up-regulation of BDNF and NT3 mRNA and protein levels in the spinal cord [94]. Increased levels of BDNF-associated mRNA and protein levels near the injury site have also been reported following treadmill training or running [94]. Additionally, direct stimulation of hindlimb stepping was noted after intrathecal administration of BDNF [95]. Other factors related to BDNF activity, such as synapsin 1, NT-3 and cyclic AMP (cAMP) response element binding protein (CREB), are also elevated in response to post-injury exercise [96].

Although injury alone may induce an increase in the levels of these neurotrophic agents [97], these astrocyte-based increases were transient (< 2 days after SCI), and thus, unlikely to facilitate later phase neuroplasticity or functional recovery. In contrast, animals that were exercised in the post-injury period demonstrated prolonged elevation of these factors, which may offer a new therapeutic window for other interventions. There is currently no agreed-upon conclusion regarding the origin of exercise-enhanced BDNF expression despite numerous studies that implemented NSCs, astrocytes, neurons and blood-born cells as neurotrophin providers under varied situations [98]. Finally, the spinal cord's inherent regenerative potentials can also be directly stimulated by post-injury physical activity. Exercise has been shown to double the number of proliferative cells in the CNS [74,99] and to have additional benefits, such as enhanced long-term potentiation of post-synaptic efficiency [100].

Overall, experimental outcomes suggest that synapse formation and maintenance can be activity-dependent (e.g., exercise-driven), largely operated by neurotrophins [101]. Such neurotrophic drives may not only promote birth of new neurons, but also facilitate oligodendrogenesis [102]. We therefore speculate that the amplitude of NSC-based release of neurotrophic factors could be further modified by physical activity-related rehabilitation modalities and specific pharmacological treatments augmenting NSC participation and their trophic factor production that, by mimicking developmental processes, augment reciprocal therapeutic relationships with surrounding cells and cue molecules [7]. 

## MECHANISMS THAT ENABLE THERAPEUTIC DELIVERY OF NEUROTROPHIC FACTORS FROM PROTOTYPE OR GENETICALLY ENGINEERED NSCS FOR SPINAL CORD REPAIR

It has been well recognized that NSCs have an innate ability to detect pathologic targets for trophic actions. We and other investigators previously described a critical feature of prototype NSCs that is distinct from non-NSC carriers of neurotrophin transgenes – the ability to detect pathological loci and adopt appropriate developmental initiatives toward customized trophic support or cell replacement [103,104]. These abilities were first suggested by experimental data that was derived from a clone of murine NSCs after their implantation into an adult rat spinal cord after segmental hemisection (i.e., open wound lesion), where the NSCs contibuted markedly to functional restoration [20]. Moreover, when NSCs of the same lineage were transplanted into adult mouse neocortex in which pyramidal neurons of a circumscribed region were induced experimentally to undergo apoptosis, they differentiated differently than when grafted into uninjured neocortex: they preferentially differentiated into pyramidal neurons, whereas these same NSCs yielded mostly glia in normally developed neocortex [103]. These data indicate that donor NSCs can respond to biological cues in normal and abnormal microenvironments, suggesting that NSCs spontaneously have mechanisms compensating for genetic [17,105] or acquired deficiencies [14]. Though the exact mechanisms regulating the capacity of NSCs to exert developmental functions in an adult microenvironment are still unknown, it is clear that when confronted with injury or other pathologic conditions, NSCs recapitulate certain of their developmental mechanisms [24,93,103].

It is currently believed that the signals modifying exogenous and endogenous NSC production of trophic factors affect a complex group of mitogens and chemokines, trophic and tropic agents, plus adhesion and extracellular matrix molecules (i.e., common developmental signaling molecules), as well as chemotactic and angiogenic factors, activated microglia, inflammatory cells, invading macrophages and damaged neurons and glia (i.e., general inflammation-mediating components). Optimization of therapy for tissue protection and repair in the post-mitotic environment of the adult CNS may require overcoming an insufficient supply of NSCs and endogenous neurotrophic factors. Hence, donor NSCs become attractive to consider as therapeutic supplements. However, exogenous prototype NSCs without engineered enhancements are vulnerable to same insults from secondary injury molecules as endogenous cells [20,106]. Therefore, we and our colleagues at the Langer Lab at MIT recently devised a novel chemical engineering approach to protect donor NSCs by applying antioxidant drug-releasing polymer film to alter the implantation *milieu* against neuroinflammation mediated by reactive nitrogen species (RNS). Our technology has been fruitful not only in enhancing the survival of exogenous NSCs but also in maintaining their capacity to produce neurotrophic factors such as GDNF and BDNF [107]. 

## APPLICATION OF NTFS OR NSCS TO TREAT NEURODEGENERATIVE DISEASES 

As a consideration of translational initiatives beyond SCI, the following sections will briefly discuss the possibility of using NTFs and NSCs in treatment of neurodegenerative diseases, citing studies on Alzheimer’s Disease (AD) and amyotrophic lateral sclerosis (ALS) as representative examples.

The potential use of NGF, a member of the neurotrophin family, for AD therapy has been reviewed in detail [29]. NGF emerged as a candidate therapeutic molecule for neuronal degeneration soon after its discovery [111], due to its role as a vital survival factor for sensory and sympathetic neurons during development [112]. NGF also prevents both lesion-induced and spontaneous atrophy of basal forebrain cholinergic neurons, as well as ameliorating memory deficits in aged rats; both are hallmark therapeutic targets for AD [113-115]. In AD animal models NGF treatment reduced neuronal death, and simultaneously stimulated cholinergic neurons, despite exposure to a cohort of deleterious mechanisms that cause neuronal degeneration, including excitotoxicity, aging, and amyloid overproduction [116-122]. NGF treatment improved cognition in a variety of models of rodent memory dysfunction, and its neuroprotective actions have been also confirmed in non-human primate models [123-128]. Mechanistically, NGF activates Erk/MAP kinase to influence a number of growth and function-related intracellular pathways *via *TrkA receptors, and activates pro-survival gene expression and inhibits apoptosis *via *the Akt pathways [127]. The studies to date validate the essential trophic impact of NGF on adult cholinergic neurons, and corroborate that NGF is needed (and produced) throughout life in the neocortex and hippocampus [128,129]. 

Therefore, if the expressions of p75 and TrkA receptors remain sufficient for NGF responsiveness, NGF delivery near degenerating cholinergic neurons appears to be an appealing clinical strategy [130-133]. However, effectively delivering NGF to the brain in humans remains a challenge, primarily because the NGF protein is too large to cross the blood brain barrier (BBB). Intra-ventricular infusion can bypass the BBB in animal models, but then, NGF spreads non-selectively in the CNS, resulting in unwanted side effects such as weight loss [134], sympathetic axon sprouting [135] and neuropathic pain related to Schwann cell invasion [136,137], despite effectiveness in the rescue of degenerating cholinergic neurons. The adverse effects were also observed in human trials [138]. Similar effects occurred in trials of patients with Parkinson’s disease when GDNF or BDNF were infused intraventricularly [139,140]. These side effects observed in clinical trials highlight the necessity to establish new approaches that can overcome certain ineffective aspects of direct NTF delivery that lacks feedback regulatory mechanisms.

ALS is a fatal neurodegenerative disease with a signature pathology of progressive motor neuron death in both the spinal cord and brain, which culminates in rapid loss of muscle function and eventual respiratory failure [141,142]. The only clinically available therapy to date is riluzole (Rilutek), which marginally extends survival by limiting excitotoxicity [143] and increasing neurotrophin release from astrocytes [144]. The majority of ALS cases are sporadic, whereas only 10% are familial (FALS) [145], among which 15-20% can be traced to point mutations in cytosolic Cu^2+^/Zn^2+^ superoxide dismutase 1 (SOD1) [146]. 

The rapid neuronal degeneration in ALS deems neuronal replacement and neuroprotection as valuable clinical approaches to treatment. Therefore, different types of progenitor or stem cells with capacity to produce NTFs have been tested in rodent models of ALS [147-151]; these include hNT neurons derived from the human teratocarcinoma cell line, mouse Sertoli cells [147,149], human umbilical cord blood cells [147,151], human [151], or mouse [148] bone marrow transplants, [152] and mouse or human NSCs [153,154]. 

Data in published studies suggests, at first glance, that the lineage or site of origin of NSCs may be responsible for some of the drastic differences in therapeutic efficacy. For instance, transplantation of human NSCs isolated from fetal forebrain (i.e., HFB2050) [14] into multiple loci of the spinal cord resulted in marked increase of life span in SOD1 (G93A) mice [154], whereas neural progenitor cells (NPCs) derived from human cortex and spinal cord produced lesser results [155]. However, further analysis suggests that the NTF profiles of donor cells might have played a key role determining the therapeutic effect in rodent ALS models [154,156]. Prototype human NSCs (HFB2050) secrete a spectrum of NTFs, including BDNF and GDNF [14], whereas genetically engineered NPCs may produce high levels of one specific NTF only, which may not be adequate to counteract ALS pathophysiology that is triggered by multiple pathogenic pathways [141]. Likewise, direct expression of GDNF delivered by lentiviral vector transfection to the lumbar spinal cord showed no beneficial effects on motor neuron survival [157], nor did robust GDNF production in the spinal cord by genetically modified NPCs prevent muscle atrophy in SOD1 rats [158]. These results underscore the need to define the range of signaling mechanisms governing the interaction between stem cells and the surrounding microenvironment to reach homeostasis by regulative mechanisms such as NTFs, other secreted molecules, and direct cell-to-cell communication (e.g., *via *gap junctions). Better understanding of these processes will ultimately enable us to design combinatorial approaches with stem cell-mediated NTF therapy to tackle complex systemic pathologies such as ALS.

To the best of our knowledge, the only controlled ALS clinical trial with stem cells reported so far was performed in Italy [159], though there had been a few uncontrolled clinical studies with other growth factors, including peripheral injection, intraventricular infusion, or intrathecal injection of CNTF and BDNF [160,161]. In the phase 1 trial the effects of autologous bone marrow derived mesenchymal stem cells (MSCs) were transplanted safely in the thoracic spinal cord of 10 ALS patients [159], and no adverse effects were seen in the 24-month post-transplantation observation period. However, no significant changes in disease progression were observed. Based on recent findings in experimental animals, glial pathology in ALS should targeted in future trials [162]. For example, ALS model mice with conditionally deleted astrocytes expressing mutant hSOD1 showed delayed disease progression [163]. By introduction of stem cells with diversified capacities to express NTFs, such as GDNF, BDNF, IGF-I or VEGF, as well as forming direct cell-to-cell communications (e.g., *via *gap junctions) [8], motor neuron survival and function may be improved through modificaion of the tissue microenvironment [109,154]. 

## FUTURE DIRECTIONS 

### Engineering NSCs as Transgene Carriers or Biopumps

It has been well established that NSCs constitutively produce a broad range of appropriately functioning peptidergic neurotrophic and neurite outgrowth-promoting factors [104]. To augment such capabilities further, NSCs have been engineered to express various transgenes, including NT-4/5, GDNF, BDNF, NGF, L1, sonic hedgehog, wnt-1, wnt-3a, as well as an assortment of biosynthetic and metabolic enzymes. Customized implantation of genetically engineered NSCs has been used to enhance neuronal differentiation, neurite outgrowth and connectivity within diseased tissue loci [108,109]. Also, NSCs have been manipulated to express the specific TrkC receptor of a neuron-inducing neurotrophin, NT-3 [33,77]. The engineered NSCs respond to NT-3 in an autocrine or paracrine fashion [33], which appears to trigger a significantly higher percentage of differentiating neurons in the TrkC-expressing NSCs group exposed to NT-3 than in the rAd-LacZ control cell group similarly exposed to NT-3 [33]. 

However, attempting to intervene in the natural expression of the various neurotrophic factors in their various proportions through genetic manipulation actually appeared to cause profile changes in trophic factor expression by the engineered cells [110]. For example, enhancing expression of NT-3 in a given clone of NSCs actually extinguished the clone’s expression of GDNF, replacing the promotion of motor axon ingrowth with an enhanced ingrowth of sensory axons [110]. Caution is called for when testing a therapeutic strategy based on transgene-mediated NTF overexpression in NSCs, for unintended effects may result from such (unanticipated) “disturbance” in post-engineering biological programs of the donor stem cells. The aforementioned facts, overall, suggest the feasibility of using *bona fide* NSCs or genetically engineered NSCs to serve as biopumps for a broad selection of biofactors when one is aiming to repair damaged or dysfunctional neural tissues.

### Induced Pluripotent Stem Cells (iPS Cells)

The past few years have seen the swift development in pluripotent stem cell technology led by the initiating report from the Yamanaka laboratory [165], in which a group of stemness- or mitosis-related transcription factor genes (Oct4, Sox2, Klf4 and c-Myc) were introduced to transform somatic cells dramatically into stem cell-like primitive entities with pluripotency and differentiation flexibility that are, to certain degrees, similar to ESCs. However, there are serious concerns regarding the safety of such iPSCs, especially the tumorigenic consequence, which currently prevents practical clinical use of iPS cells. Significant intrinsic variability in the derived iPSCs, abnormal expression of imprinted genes due to the random integration of transcription factors, persistent donor gene expression, and difficulty in obtaining a sufficiently rapid cell multiplication rate *in vitro* to build up a cell population sufficient for use in human patients further limit the clinical use of these cells [165-167]. Therefore, additional molecular strategies using alterative gene targets [168,169], fewer targets [170-173], or viral vector-free transfection technologies have been developed [175-178], to circumvent the safety deficits of iPS cells before their therapeutic potential can be systematically examined. However, recent reports showed that patient-specific iPSCs can be generated for investigative studies on drug screening or for experimental therapy trials [179-181]. In addition, direct trans-differentiation of adult cell types has been presented as a further therapeutic option for reversing disease progression or transforming neighboring healthy cells to take over functions lost by diseased or dying cells [182]. Despite these advances, there appear to be no reports to date on NTF production by iPS cells, a pivotal parameter of stem cell biology [7], albeit there is recent important progress in the use of synthetic modified mRNA to increase efficiency of cell reprogramming and directed differentiation, as well as analyses of the impact of epigenetic memory on the molecular and functional properties of iPS cells [183-186].

## A SUMMARY NOTE

**Fig. (1):**
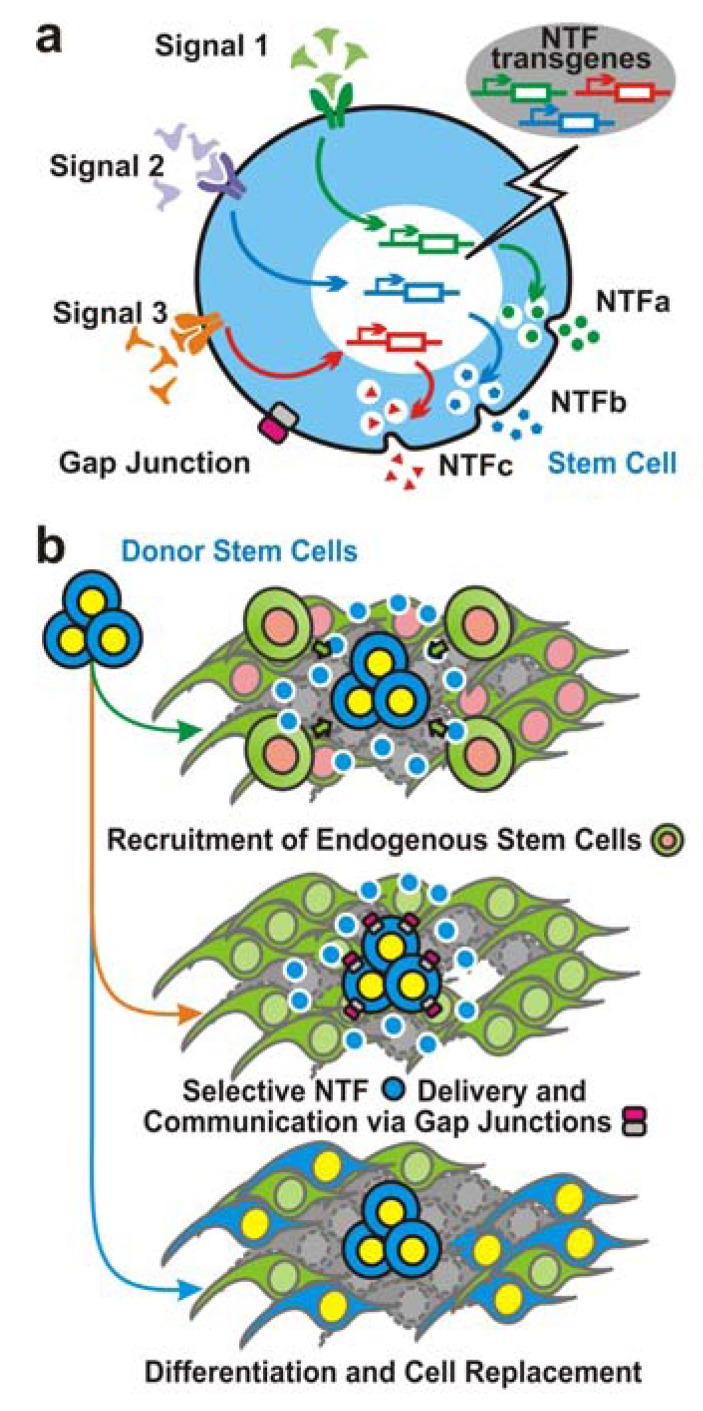
**Schematic summary of stem cell-based therapeutic strategies. a,** Stem cells possess intrinsic trophic factor producing abilities, and are able to respond to environmental cues to customize the profile of trophic factor production to stage homeostasis, which, as a capacity, can be further augmented by genetically engineering the cells with extra copies of transgenes of desirable molecules. **b**, Donor stem cells, prototype or genetically modified, may provide therapeutic benefits through at least three distinct mechanisms that could cast synergistic impacts: (1) replacement of the dysfunctional or dead host cells; (2) homeostatic regulation through delivery of trophic factors in targeted manners that are biologically regulated (e.g., in response to particular environmental cues, etc.) as well as establishment of gap junctions (**b**: upper inset), etc.; and (3) recruitment of and nourishment for host endogenous stem cells. The aforementioned therapeutic mechanism No. 2, apparently, carries a wide spectrum of regulatory tactics that can be further explored to refine the trophic factor and/or other cytokine secretion at each developmental stage or neural disorder status as NSCs integrate into and prepare, modify, and guide the surrounding CNS environment towards the formation and homeostatic maintenance of a physiologically functioning adult nervous system. We believe that effectively adopting this conceptual view of the stem cell-based NTF application is essential for correct, efficient, and optimal use of trophic factors to reach their designated therapeutic goals.

Academia and the public have, in general, accepted the concept that stem cells provide an enormous opportunity to advance the understanding of developmental biology, and especially to develop novel clinical therapies. However, we still need refined knowledge on how NSCs can be crafted to deliver the range of NTFs that would meet the micro-environmental requirements to establish homeostasis that would offset an ongoing disease process. This ability of stem cells, buoyed by their innate developmental biological properties, may teach us how to mitigate the adverse side effects resulting from direct administration of NTFs one at a time. Complex diseases such as ALS, AD and Parkinson’s disease are especially likely to require complex solutions. NSCs, as described in this review, can interface and work synergistically with gene and growth factor therapy, gap junction-mediated homeostasis, anti-apoptotic and neuroprotective strategies, stimulation of neurogenesis, anti-inflammatory and anti-scarring approaches, material science and tissue engineering, and at least in the case of SCI, with physical activity and exercise. Therefore, we propose an updated concept of NTF application. The traditional approach based on the pharmacological principles of trophic factor delivery (i.e., the dose and duration of administering a particular NTF), needs be revised to incorporate the newly appreciated importance of feedback-controlled production of trophic factors by the NSCs to mediate systemic homeostasis. Under this new conceptual context, we can begin to understand the wide spectrum of molecular tactics the NSCs deploy to regulate their trophic factor secretion at each normal developmental stage as they integrate into and prepare, modify, and guide the surrounding CNS environment towards the formation and homeostatic maintenance of a physiologically functioning adult nervous system. We believe that effectively adopting this conceptual view of “functional multipotency” of stem cells is essential for correct, efficient, and optimal use of trophic factors toward therapeutic goals (Fig. **1**) [7,151].
